# BATF2 prevents glioblastoma multiforme progression by inhibiting recruitment of myeloid-derived suppressor cells

**DOI:** 10.1038/s41388-020-01627-y

**Published:** 2021-01-15

**Authors:** Xin Zhang, Yi Liu, Lei Dai, Gang Shi, Jie Deng, Qiang Luo, Qian Xie, Lin Cheng, Chunlei Li, Yi Lin, Qingnan Wang, Ping Fan, Hantao Zhang, Xiaolan Su, Shuang Zhang, Yang Yang, Xun Hu, Qiyong Gong, Dechao Yu, Lei Zheng, Hongxin Deng

**Affiliations:** 1 State Key Laboratory of Biotherapy and Cancer Center, West China Hospital, Sichuan University and Collaborative Innovation Center for Biotherapy, Chengdu, 610041 Sichuan PR China; 2 Laboratory Medicine Center, Nanfang Hospital, Southern Medical University, Guangzhou, 510515 Guangdong PR China; 3 Huaxi MR Research Center, Department of Radiology, West China Hospital, Sichuan University, Chengdu, 610041 Sichuan PR China; 4 General Practice Department of West China Hospital, Sichuan University, Chengdu, 610041 Sichuan PR China; 5 Huaxi Biobank, West China Hospital, Sichuan University, Chengdu, 610041 Sichuan PR China; 6 Cancer Center, West China Hospital, Sichuan University, Chengdu, 610041 Sichuan PR China

**Keywords:** Tumour biomarkers, Cell biology

## Abstract

The basic leucine zipper ATF-like transcription factor 2 (BATF2) has been implicated in inflammatory responses and anti-tumour effects. Little, however, is known regarding its extracellular role in maintaining a non-supportive cancer microenvironment. Here, we show that BATF2 inhibits glioma growth and myeloid-derived suppressor cells (MDSCs) recruitment. Interestingly, extracellular vesicles (EVs) from BATF2-overexpressing glioma cell lines (BATF2-EVs) inhibited MDSCs chemotaxis in vitro. Moreover, BATF2 inhibited intracellular SDF-1α and contributes to decreased SDF-1α in EVs. In addition, BATF2 downregulation-induced MDSCs recruitment were reversed by blocking SDF-1α/CXCR4 signalling upon AMD3100 treatment. Specifically, detection of EVs in 24 pairs of gliomas and healthy donors at different stages revealed that the abundance of BATF2-positive EVs in plasma (BATF2^+^ plEVs) can distinguish stage III–IV glioma from stage I–II glioma and healthy donors. Taken together, our study identified novel regulatory functions of BATF2 in regulating MDSCs recruitment, providing a prognostic value in terms of the number of BATF2^+^ plEVs in glioma stage.

## Introduction

Glioblastoma multiform (GBM) is the most common and lethal type of human primary brain tumour [1, 2]. The median survival rate of GBM patients is <16 months [3]. The tumour microenvironment (tumour cells located in the internal and external environment, TME) is vital for the occurrence, growth, and progression of tumours [4, 5]. Myeloid-derived suppressor cells (MDSCs) are a heterogeneous population of bone marrow-derived cells that accumulate during various pathological conditions, particularly in gliomas [6–8]. MDSCs release vascular endothelial growth factor A (VEGFA) and matrix metalloproteinases (MMP2 and MMP-9) to promote glioma growth [9–11]. Defining the interactions between tumour cells and the tumour microenvironment is a key in the treatment of glioma [9, 12, 13]. Importantly, targeting tumour-associated MDSCs in the microenvironment is an alternative therapeutic strategy for glioma [9, 14–16].

Extracellular vesicles (EVs) include small Extracellular vesicles (sEVs) with a size range of approximately 40–160 nm, and microparticles, microvesicles, large vesicles with diameters ranging from 50 nm to 1 μm [17, 18]. EVs are formed by inward and outward budding of endosomes and ectosomes, resulting in encapsulation of nucleic acids and proteins [19, 20]. Tumour-derived EVs are known to play key roles in communication between tumour cells and the tumour microenvironment [21, 22]. An increasing number of studies have shown that EVs are closely related to the tumour microenvironment [22, 23]. Bioactive molecules from tumour cells carried by EVs contact and activate tumour-promoting cells (e.g., MDSCs and Tumour-associated macrophage, TAMs) eventually [18, 19, 24]. EVs can be effective circulating markers that reflect tumour progression, playing an important role in the field of liquid biopsy [25, 26]. Hence, in recent years, EVs in plasma (plEVs) have been identified as circulating biomarkers capable of diagnosing glioma and other cancer types [23, 27].

Basic leucine zipper ATF-like transcription factor 2 (BATF2), also known as suppressor of AP-1 regulated by interferon (SARI), reduces *CCN1* promoter activity by inhibiting AP-1 binding [28, 29]. Recent studies have reported that BATF2 overexpression inhibits tumour cell proliferation and metastasis, and promotes apoptosis in various cancer types [30]. In patients, the loss of BATF2 expression facilitates epithelial-mesenchymal transition by activation of hepatocyte growth factor/c-Met signalling [31, 32]. A potential immunotherapy combination comprising a DNA methyltransferase inhibitor and BATF2 exerts an anti-tumour effect on medulloblastoma [3]. Moreover, BATF2 upregulates IL-12p40 in TAMs to induce CD8^+^ T-cell activation, which affects CD8α^+^ dendritic cell development and B lymphoma cell killing [30]. A previous study from our group demonstrated that BATF2 targets ceruloplasmin to inhibit hypoxia inducible factor (HIF-1α), thereby hindering colon tumour growth [33]. However, the role of BATF2 in glioma growth and the tumour microenvironment is not completely understood.

In this study, we found that BATF2 prevents GBM growth by inhibiting MDSCs recruitment, and EVs from BATF2-overexpressing cell lines inhibit MDSCs recruitment in vitro. Furthermore, digital EVs detection in 24 pairs of glioma plasmas at different stages indicated that plasma BATF2^+^ EVs can constructively distinguish stage III–IV glioma from stage I–II glioma and healthy donors. Of note, our novel findings established the regulatory functions of BATF2 and BATF2-EVs in regulating MDSCs and proposed the potential of BATF2^+^ plEVs as a biomarker to reflect glioma stage.

## Results

### BATF2 overexpression inhibits GBM tumourigenesis

**Fig. 1:**
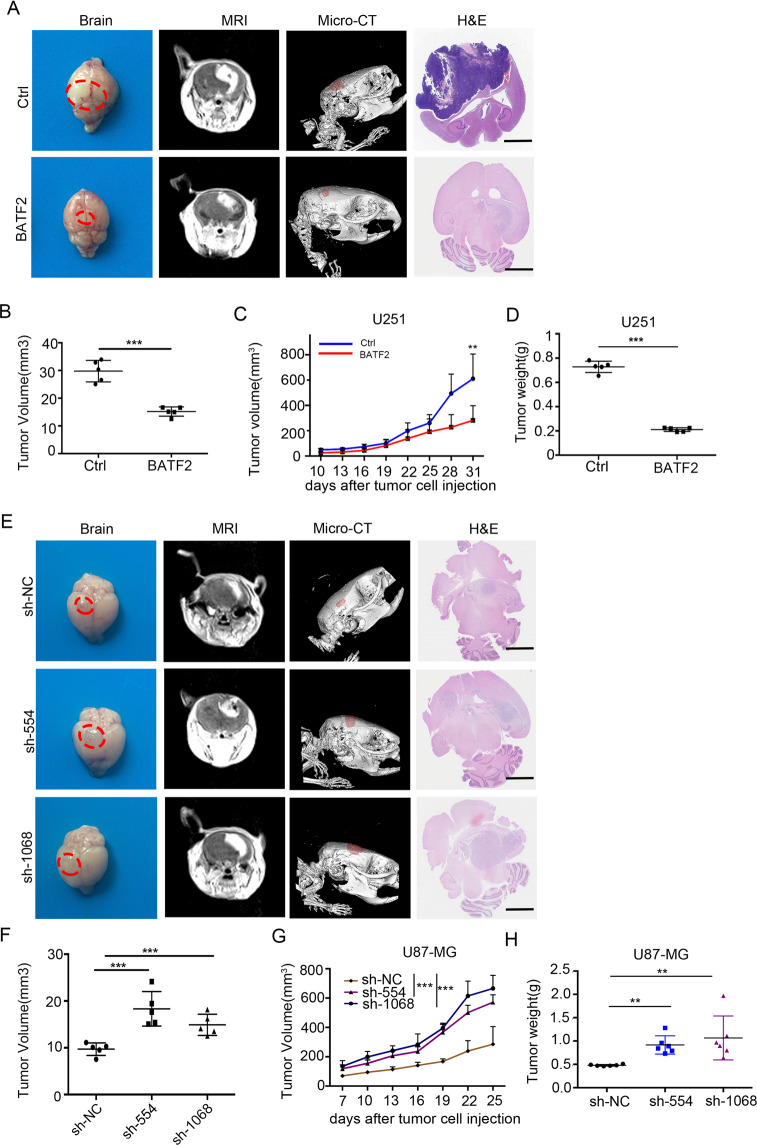
BATF2 overexpression inhibits GBM tumourigenesis. **A** Representative tumour, MRI images (35 days), 3D-Micro-CT images (35 d), and H&E (35 d) of U251-Ctrl and U251-BATF2 group. Red dotted area highlights tumour area. Scale bars,100 μm. **B** The average intracranial tumour volume (*n* = 5, *p < 0.05, **p < 0.01, ***p < 0.001). **C** The average subcutaneous tumour volume of U251-Ctrl and U251-BATF2 (*n* = 5, *p < 0.05, **p < 0.01, ***p < 0.001). **D** The average subcutaneous tumour weight of U251-Ctrl and U251-BATF2 (*n* = 5, *p < 0.05, **p < 0.01, ***p < 0.001). **E** Representative tumour, MRI images, 3D-micro-CT images, and H&E images of U87-sh-NC, U87-sh-554 and U87-sh-1068 group. Red dotted area highlights tumour area. Scale bars,100 μm. **F** The average intracranial tumour volume (*n* = 5, *p < 0.05, **p < 0.01, ***p < 0.001). **G** The average tumour volume of U87-sh-NC, U87-sh-554 and U87-sh-1068 (*n* = 6, *p < 0.05, **p < 0.01, ***p < 0.001). **H** The average tumour weight of U87-sh-NC, U87-sh-554 and U87-sh-1068 (*n* = 6, *p < 0.05, **p < 0.01, ***p < 0.001).

To address the functional association of BATF2 in glioma formation, GBM cells were selected for intracranial tumourigenesis assays in BALB/c nude mice. We detected BATF2 expression at both the mRNA and protein levels in a human astrocyte cell line (HA1800) and five distinct glioma cell lines (U251, U87-MG, LN-18, U118-MG, and A172). The results revealed diminished BATF2 expression in U251 cells and elevated expression in U87-MG cells (Fig. S1A, B). Thus, we infected U251 cells with lentivirus containing either an empty vector (LV-Ctrl) or a vector encoding human BATF2 (LV-BATF2). In addition, we also infected U87-MG cells with 2 types of lentivirus-encoded shRNA targeting *BATF2* (sh-1068 and sh-554) or control shRNA (sh-NC). BATF2 expression after viral transduction was confirmed by western blotting and qPCR analyses (Fig. S1C, D). Next, we orthotopically implanted 1 × 10^5^ U251-Ctrl and U251-BATF2 cells in the forebrain of nude mice and performed MRI and micro-CT detection to assess initial tumour growth (Fig. S2). 3D video imaging showed an established glioma model (Movie S1). Five weeks after tumour implantation, we found that overexpression of BATF2 in U251 cells significantly inhibited tumour growth (red dotted lines highlight the tumour regions) (Fig. 1A). Tumour volumes (*n* = 5) were measured at the end point (*p* < 0.001) (Fig. 1B). Furthermore, subcutaneous injection of U251-Ctrl and U251-BATF2 cells demonstrated that upregulation of BATF2 significantly reduced tumour volume and weight by ~64.8% (*p* < 0.01) (Fig. 1C, D). In contrast, MRI scanning and H&E staining of U87-sh-BATF2 downregulation xenografts showed that tumour masses were significantly increased when BATF2 was downregulated (red dotted lines highlight tumour areas) (Fig. 1E, F). Tumour volume and weight statistics confirmed the same results (*p* < 0.001) (Fig. 1G, H). Collectively, these data confirmed that BATF2 inhibits tumour growth in both intracranial and subcutaneous GBM models.

### BATF2 inhibits recruitment of monocyte-derived MDSCs to tumour microenvironment

**Fig. 2:**
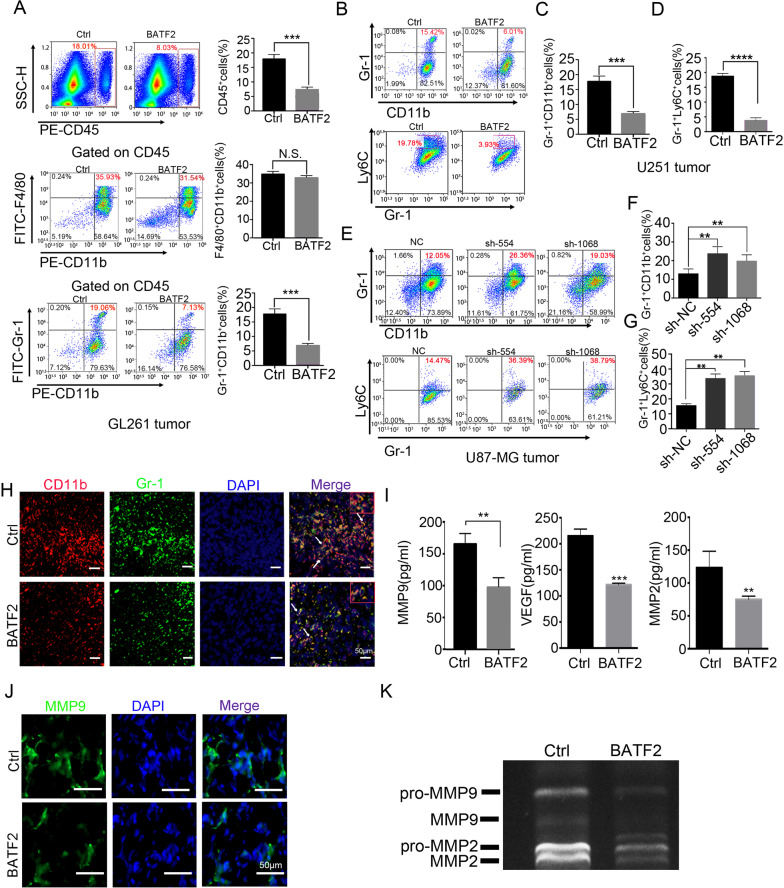
BATF2 inhibits the recruitment of monocyte-derived MDSCs to tumour microenvironment. **A** FACs analysis and statistics of BMDMs in GL261-Ctrl and GL261-BATF2 subcutaneous tumours after 15 days injection (*n* = 3, independent experiment, *p < 0.05, **p < 0.01, ***p < 0.001). **B** FACs analysis of GR-1^+^CD11b^+^MDSCs and Ly-6C^+^Gr-1^+^Mo-MDSCs/MDSCs in U251-Ctrl and U251-BATF2 after 15 days injection. **C** Statistics of CD11b^+^Gr-1^+^ MDSCs in U251-Ctrl and U251-BATF2 after 15 days injection (*n* = 3, independent experiment, *p < 0.05, **p < 0.01, ***p < 0.001). **D** Statistics of Gr-1^+^Ly-6C^+^ Mo-MDSCs/CD11b^+^Gr1^+^ MDSCs in U251-Ctrl and U251-BATF2 after 15 days injection (*n* = 3, independent experiment, *p < 0.05, **p < 0.01, ***p < 0.001). **E** FACs analysis of CD11b^+^Gr-1^+^MDSCs and Gr-1^+^Ly-6C^+^Mo-MDSCs/CD11b^+^Gr-1^+^ MDSCs in U87-sh-NC, U87-sh-554 and U87-sh-1068 after 15 days injection. **F** Statistics of CD11b^+^Gr-1^+^ MDSCs in U87-sh-NC, U87-sh-554 and U87-sh-1068 after 15 days injection (*n* = 3, independent experiment, *p < 0.05, **p < 0.01, ***p < 0.001). **G** Statistics of Gr-1^+^Ly-6C^+^ Mo-MDSCs/CD11b^+^Gr-1^+^ MDSCs in U87-sh-NC, U87-sh-554 and U87-sh-1068 after 15 days injection (*n* = 3, independent experiment, *p < 0.05, **p < 0.01, ***p < 0.001). **H** Representative images of immunofluorescent staining with Gr-1 (green), CD11b (red) and DAPI (blue) in U251-Ctrl and U251-BATF2 intracranial tumours. White triangles indicate the MDSCs. Scale bars, 50 μm. I. ELISA detection of MMP-9, MMP2, VEGF in U251-Ctrl and U251-BATF2 intracranial tumours (*n* = 3, independent experiment, *p < 0.05, **p < 0.01, ***p < 0.001). **J** Representative images of immunofluorescent staining with MMP-9 (green) and DAPI (blue) in U251-Ctrl and U251-BATF2 intracranial tumours. Scale bars, 50 μm. **K** Zymogram analysis of U251-Ctrl and U251-BATF2 tumours.

Next, we investigated how BATF2 suppresses tumour growth. Interestingly, Cell Counting Kit-8 analyses demonstrated that both overexpression and downregulation of BATF2 in U251, U87-MG, U118-MG, and A172 glioma cells did not affect cell viability in vitro (Fig. S3A–D). Furthermore, BATF2 did not affect invasion and migration by tumour cells, as shown by transwell migration assay in vitro (Fig. S3E–H). In addition, immunohistochemical (IHC) staining for proliferating cell nuclear antigen (PCNA) in subcutaneous tumour tissues indicated that upregulated BATF2 expression did not inhibit tumour cell proliferation (Fig. S4A). IHC staining for PCNA in the U87-sh-554, U87-sh-1068, and U87-sh-NC groups confirmed these results (Fig. S4B). Therefore, we hypothesised that BATF2 may not directly regulate glioma cell proliferation, but affects the tumour microenvironment instead. Herein, we established glioma model using a mouse glioma cell line, GL261, and stable cell lines, GL261-BATF2, and found that overexpression of BATF2 inhibited GL261 subcutaneous tumour growth, as expected. By calculating the percentages of bone marrow-derived cell (CD45^+^, mainly including MDSCs and macrophages) recruitment into tumour (Fig. S5), we found that upregulation of BATF2 significantly inhibited CD45^+^ cell infiltration (*p* < 0.001). Furthermore, we observed a significant decrease in the percentage of Gr-1^+^CD11b^+^ MDSCs gated on CD45^+^cells in GL261-BATF2 compared to GL261-Ctrl (*p* < 0.05). However, there was no significant change in F480^+^CD11b^+^ TAMs (Fig. 2A). We further stained MDSCs using a monocyte marker (Ly-6C) and a granulocyte marker (Ly-6G)(data not shown), and found that upregulation of BATF2 caused a significant reduction in Ly-6C^+^Gr-1^+^monocytes-MDSCs (Mo-MDSCs) and MDSCs recruitment in human glioma cell line U251-BATF2 compared to U251-Ctrl (*p* < 0.001) (Fig. 2B–D). In addition, a significant increase in Mo-MDSCs and MDSCs recruitment into tumour tissues was observed in the U87-sh-554 and U87-sh-1068 groups compared to U87-sh-NC (Fig. 2E–G). Moreover, our findings were also supported by immunostaining (Fig. 2H). In human GBM, VEGFA, MMP-9, and MMP2 levels correlate with glioma progression and could be secreted by both tumour cells and MDSCs [11]. We also performed ELISA detection and found that BATF2 upregulation significantly decreased VEGFA, MMP2, and MMP-9 levels in glioma tissues (VEGFA: *p* < 0.001; MMP2: *p* < 0.01; MMP-9: *p* < 0.01; Fig. 2I) and exhibited decreased MMP-9 staining in U251-BATF2 tumour (Fig. 2J). Moreover, zymography results indicated that MMP2 and MMP-9 activity decreased in the BATF2 group (Fig. 2K). These data collectively suggested that BATF2 inhibits glioma progression and Mo-MDSCs infiltration.

### EVs from BATF2-overexpressing cell lines inhibit MDSCs chemotaxis in vitro

**Fig. 3:**
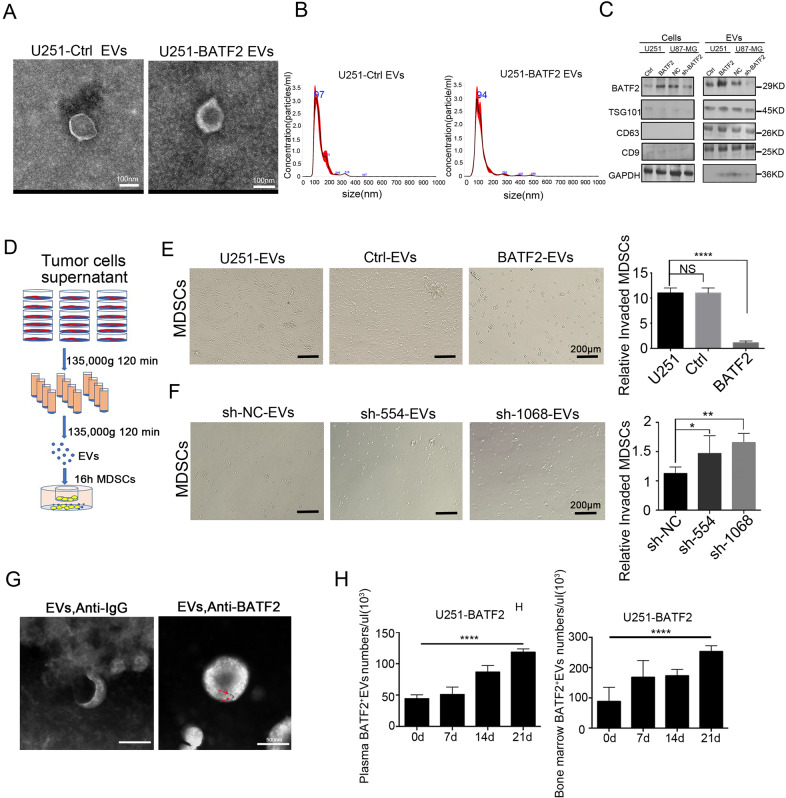
EVs from BATF2-overexpressing cell lines inhibit MDSCs chemotaxis in vitro. **A** Images of U251-Ctrl and U251-BATF2 derived EVs from supernatant were taken by scanning Transmission electron microscopic analysis. Scale bars, 100 nm. **B** Nanoparticle tracking analysis results from representative U251-Ctrl and U251-BATF2 derived EVs samples are shown (1:100 dilution with particle-free PBS). **C** The protein levels of BATF2, TSG101, CD9, CD63, and GAPDH in U251-Ctrl, U251-BATF2, U87-sh-NC, U87-sh-BATF2 tumour cells-derived EVs and tumour cells were assessed by western blotting. **D** Experimental design for Matrigel invasion assay of tumour-derived EVs activated and recruiting MDSCs. **E** Representative images and statistics of invaded MDSCs cells after co-culture with U251, U251-Ctrl, U251-BATF2 tumour-derived EVs. Scale bars, 200 μm (*n* = 3, independent experiment, *p < 0.05, **p < 0.01, ***p < 0.001). **F** Representative images and statistics of invaded MDSCs cells after co-culture with U87-sh-NC, U87-sh-554, U87-sh-1068 tumour-derived EVs. Scale bars, 200 μm (*n* = 3, independent experiment, *p < 0.05, **p < 0.01, ***p < 0.001). **G** Transmission electron microscopy of GBM patient plasma EVs stained with primary anti-BATF2 and gold-conjugated secondary antibody. The arrow indicates EV staining positive for BATF2. Scale bars, 100 nm. **H** Exo-Counter detection of BATF2^+^EVs in plasma and bone marrow of U251-BATF2-bearing mice in 0, 7, 14, 21 days (*n* = 3, independent experiment, *p < 0.05, **p < 0.01, ***p < 0.001).

Tumour-derived EVs play key roles in communication between tumour cells and the microenvironment [34]. Thus, we hypothesised that MDSCs inhibition caused by BATF2 was related to EVs. To resolve this conjecture, we isolated EVs from U251-Ctrl and U251-BATF2 cell lines. Transmission electron microscopy (TEM) studies showed that tumour-derived EVs exhibited similar size distribution and morphology in U251-Ctrl and U251-BATF2 cells (Fig. 3A). In addition, nanoparticle tracking analysis (NTA) of EVs from tumour cell supernatants also showed comparable size distributions in U251-Ctrl and U251-BATF2 cells, with a mode size, respectively (Fig. 3B). The particle numbers of depleted EVs from foetal bovine serum were also confirmed by NTA analysis (Fig. S6A). Furthermore, the expression of EVs markers (CD63, CD9, TSG101), GAPDH and BATF2 were detected in U251-Ctrl, U251-BATF2, U87-NC and U87-sh-BATF2 supernatant-derived EVs and cells by western blotting. These data showed that EVs concentrated from U251-BATF2 showed higher BATF2 protein levels than those from U251-Ctrl cells. In addition, decreased BATF2 was detected in EVs released from sh-BATF2 cell lines compared to sh-NC (Fig. 3C). To further provide in vitro evidence for the role of BATF2-EVs in inhibiting MDSCs chemotaxis, we isolated splenic MDSCs from GL261-bearing BALB/c nude mice (Fig. S6B) and seeded EVs from BATF2-overexpressing or -knockdown cells in the bottom of plate wells and incubated them with freshly isolated splenic MDSCs in the top chambers for 16 h (Fig. 3D). By counting the number of MDSCs that invaded through the membrane, we found that MDSCs numbers at the bottom significantly decreased when co-cultured with BATF2-EVs, compared to the Ctrl-EVs group (Fig. 3E). In contrast, EVs from BATF2-downregulated cell lines (U87-sh-554 and U87-sh-1068) promoted MDSCs chemotaxis in vitro (Fig. 3F). The exo-counter was used to detect surface proteins in the EVs by combining with nano-beads and without purification [35] (Fig. S6C, D). Thus, GBM-derived EVs incubated with gold-conjugated BATF2 antibody were scanned by TEM. The results showed that there were BATF2 proteins on the EV surface, which could be labelled (Fig. 3G). Thus, we counted the number of BATF2^+^ EVs in plasma bone marrow of U251-BATF2 tumour-bearing mice by using the Exo-counter platform. We observed that when BATF2 was overexpressed in U251 cells, BATF2^+^EVs were detected in the plasma and bone marrow of U251-BATF2-injected mice over time (Fig. 3H). These data showed that when BATF2 was overexpressed in tumours, BATF2^+^ EV concentrations in plasma and bone marrow of tumour-bearing mice may be upregulated in vivo. Taken together, our results suggested that EVs from BATF2-overexpressing cells effectively inhibit MDSCs recruitment.

### BATF2 inhibits SDF-1α expression and CXCR4-positive MDSCs infiltration in intracranial tumours

**Fig. 4:**
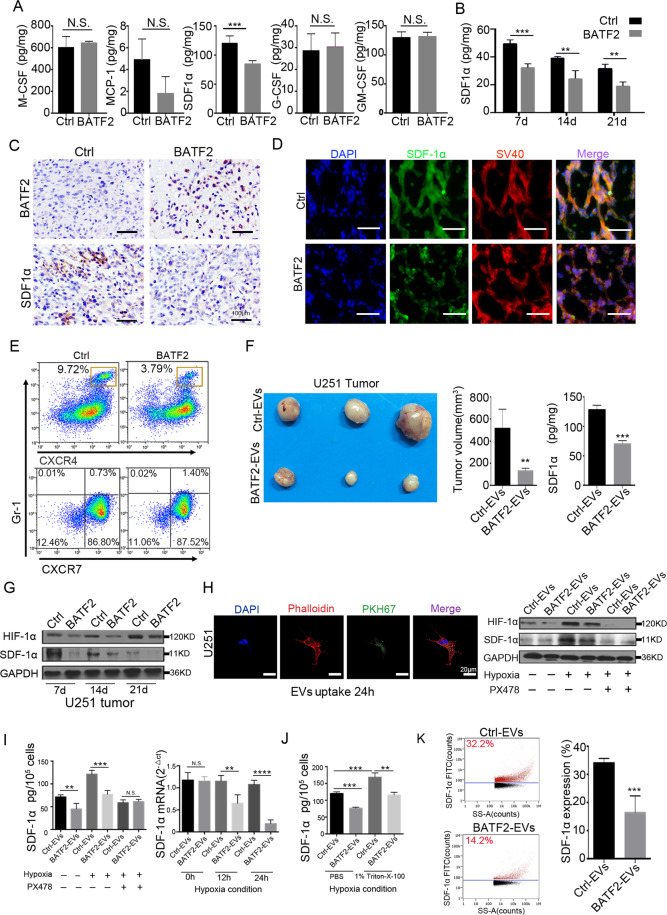
BATF2 inhibits SDF-1α expression and CXCR4-positive MDSCs infiltration in intracranial tumours. **A** ELISA detection of MDSCs-associated cytokines in U251-Ctrl and U251-BATF2 tissues (*n* = 3, independent experiment, *p < 0.05, **p < 0.01, ***p < 0.001). **B** ELISA detection of SDF-1α in 7, 14, and 21 days in U251-Ctrl and U251-BATF2 tumour (*n* = 3, independent experiment, *p < 0.05, **p < 0.01, ***p < 0.001). **C** Representative IHC images of BATF2 and SDF-1α on consecutive sections of U251-Ctrl and U251-BATF2 intracranial tumours. Scale bars, 100 μm. **D** Representative images of immunofluorescent staining with SDF-1α (green), SV40 large T (red) and DAPI (blue) in U251-Ctrl and U251-BATF2 intracranial tumours. Scale bars, 25 μm. **E** Percentages of CXCR4 and CXCR7 expressing cells in CD11b^+^Gr1^+^ MDSCs isolated from tumours by FACs analysis. **F** Representative image of U251 tumour injected with U251-Ctrl- and U251-BATF2-derived EVs for four times, the subcutaneous tumour volume and SDF-1α detection by ELISA (*n* = 3 independent experiment, *p < 0.05, **p < 0.01, ***p < 0.001). **G** Western blot detection of HIF-1α and SDF-1α in 7, 14 and 21 days during U251 tumour progression. **H** EVs labelled by PKH67 added to phalloidin staining U251 cells and western blot detection of HIF-1α and SDF-1α. Scale bars, 20 μm. **I** ELISA and qPCR detection of SDF-1α in U251 tumour treated with Ctrl-EVs and BATF2-EVs (*n* = 3, independent experiment, *p < 0.05, **p < 0.01, ***p < 0.001). **J** ELISA detection of SDF-1α with and without 1% Triton X-100 under hypoxia condition (*n* = 3, independent experiment, *p < 0.05, **p < 0.01, ***p < 0.001). **K** Nano-FCM analysis and statistics of SDF-1α expression in Ctrl-EVs and BATF2-EVs (*n* = 3, independent experiment, *p < 0.05, **p < 0.01, ***p < 0.001).

Next, we performed ELISA to detect MDSCs-associated chemokines (SDF-1α, MCP-1, M-CSF, GM-CSF, and G-CSF) in tumours. Our data showed that SDF-1α levels in intracranial tumours were significantly decreased in U251-BATF2 tumour tissues *(p* < 0.001) (Fig. 4A). However, the local expression of GM-CSF, MCP-1, M-CSF, and G-CSF did not change (Fig. 4A). In particular, BATF2 upregulation inhibited the secretion of SDF-1α during glioma progression upon intracranial tumour cell injection for 7, 14, and 21d (Fig. 4B). In addition, IHC staining confirmed the inverse correlation between BATF2 and SDF-1α levels (Fig. 4C, Fig. S7A). To further confirm the reduction in SDF-1α abundance, we also co-stained intracranial tumour tissues with a glioma-specific marker SV40-Tag, along with SDF-1α [36]. The results showed that localisation of SDF-1α surrounding tumour cells was attenuated by BATF2 overexpression (Fig. 4D and Fig. S7B). Since CXCR4 is the major receptor for SDF-1α, we found that BATF2 overexpression mainly inhibited recruitment of CXCR4^+^ MDSCs (*p* < 0.01) but not CXCR7^+^ MDSCs (non-significant) (Fig. 4E). Immunofluorescence co-staining confirmed that a low number of CXCR4^+^ MDSCs were present in U251-BATF2 tumours (Fig. S7C), and very few CXCR7^+^ MDSCs were observed in both U251-Ctrl and U251-BATF2 tumours (Fig. S7D). To demonstrate whether BATF2-EVs have inhibitory effects on SDF-1α, we subcutaneously injected U251-Ctrl and U251-BATF2 supernatant-derived EVs per 3 d after 7 d of U251 tumour establishment. We noticed that an injection of EVs from U251-BATF2 supernatant significantly inhibited U251 growth (*p* < 0.001) (Fig. 4F). ELISA results indicated that injected BATF2-EVs led to significant decrease in SDF-1α levels in tumour tissues (Fig. 4F). HIF-1α pathways have been reported to increase SDF-1α expression [12, 37, 38]. We found that HIF-1α expression was activated during U251 tumour progression between d7 and d21 and was decreased in the BATF2 group at all three different stages (Fig. 4G). To prove the role of BATF2-EVs in the contribution of SDF-1α decreasing, in vitro EVs uptake in U251 cells was confirmed by confocal microscopy (Fig. 4H). Western blotting and ELISA results showed that under normoxic conditions, BATF2-EVs uptake had only a slight effect on cellular SDF-1α expression, while under hypoxia, BATF2-EVs treatment clearly inhibited HIF-1α and SDF-1α expression. Additional supply of PX478 blocked the HIF-1α pathway, preventing BATF2 or BATF2-EVs-induced SDF-1α inhibition (Fig. 4H). Furthermore, both ELISA and qPCR data confirmed that BATF2-EVs treatment inhibited SDF-1α expression under hypoxic conditions (Fig. 4I). Previous studies have shown that SDF-1α is a secretory protein [24, 36]. However, it is not clear if SDF-1α is present in EVs. Thus, we liberated EV contents with the non-ionic surfactant Triton X-100, and subsequent ELISA data showed that Triton X-100 treatment resulted in 1.5-fold upregulation in levels, whereas SDF-1α was significantly downregulated in the BATF2-EVs co-treatment group (Fig. 4J). In addition, western blotting and ELISA detection confirmed that BATF2 inhibited endogenous HIF-1α and SDF-1α in U251 cells (Fig. S8A, B). SDF-1α on the membranes of EVs was detected by TEM scanning when labelled with gold-conjugated anti-SDF-1α antibody (Fig. S8C). Furthermore, Nano-FCM analysis [2, 39, 40] revealed the percentage of SDF-1α^+^ EVs in U251-Ctrl cells (32.2%), with fewer in U251-BATF2 (14.2%) (*p* < 0.05) (Fig. 4K). These data suggested that SDF-1α levels were decreased in BATF2-EVs. Taken together, these results suggested that BATF2-induced inhibition of Mo-MDSCs recruitment may be related to SDF-1α.

### Increased MDSCs recruitment and tumour growth by BATF2-downregulation were reversed by blocking CXCR4 signalling

**Fig. 5:**
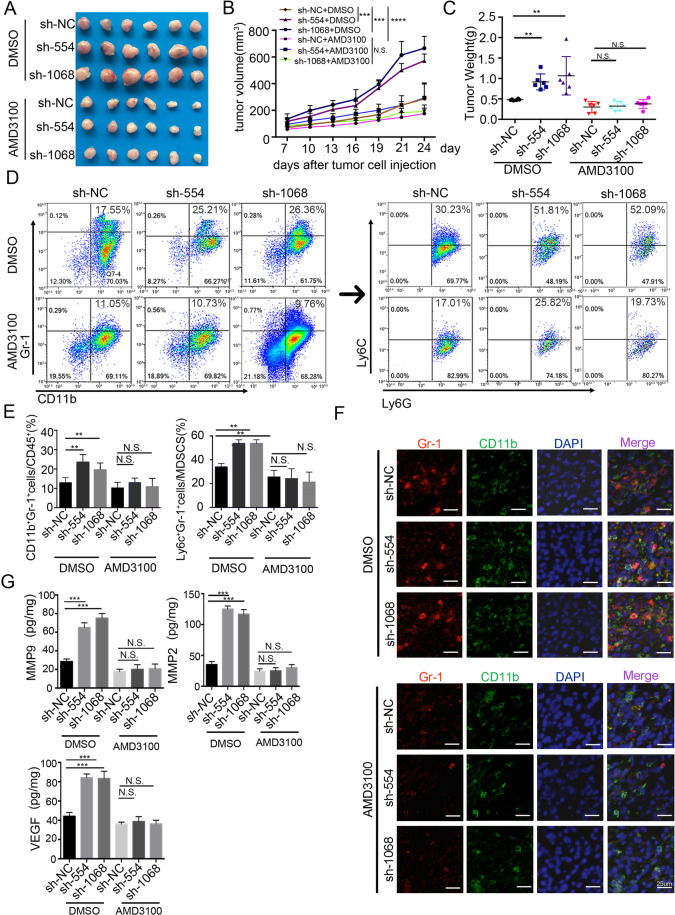
Increased MDSCs recruitment and tumour growth by BATF2-downregulation were reversed by blocking CXCR4 signalling. **A** Tumour image of DMSO and AMD3100-treated U87-sh-NC, U87-sh-554, U87-sh-1068 injected mice. B. Tumour volume of DMSO and AMD3100-treated U87-sh-NC, U87-sh-554, U87-sh-1068 injected mice (*n* = 6, *p < 0.05, **p < 0.01, ***p < 0.001). **C** Tumour weight of DMSO and AMD3100-treated U87-sh-NC, U87-sh-554, U87-sh-1068 injected mice (*n* = 6, *p < 0.05, **p < 0.01, ***p < 0.001). **D** FACs analysis of CD11b^+^Gr1^+^ MDSCs in tumours of injected with U87-sh-NC, U87-sh-554 and U87-sh-1068 treated with DMSO and AMD3100. **E** Statistics of CD11b^+^Gr1^+^ MDSCs in tumours of injected with U87-sh-NC, U87-sh-554 and U87-sh-1068 treated with DMSO and AMD3100 (*n* = 3, independent experiment, *p < 0.05, **p < 0.01, ***p < 0.001). **F** Representative images of immunofluorescent staining with Gr-1 (red), CD11b (green) and DAPI (blue) in U87-sh-NC, U87-sh-554, and U87-sh-1068 treated with DMSO and AMD3100. Scale bars, 25 μm. G. ELISA detection of MMP-9, VEGF, MMP2 in U87-sh-NC, U87-sh-554, U87-sh-1068 injected with DMSO and AMD3100 (*n* = 3, independent experiment, *p < 0.05, **p < 0.01, ***p < 0.001).

Next, we blocked SDF-1α/CXCR4 signalling by using AMD3100 [14], a CXCR4 receptor antagonist, and investigated the effects of CXCR4 blockade on tumour growth. First, we implanted U87-MG (U87-NC) and sh-BATF2-transduced U87-MG cells (U87-sh-554 and U87-sh-1068) into BALB/c nude mice subcutaneously and intratumoural injected the mice with AMD3100. As expected, downregulation of BATF2 in U87-MG cells promoted tumour growth. Interestingly, AMD3100 treatment drastically reduced tumour growth in BATF2 knock-down U87-MG cells (U87-sh-554 and U87-sh-1068) (Fig. 5A–C). In addition, we analysed MDSCs populations in U87-sh-NC-, U87-sh-554-, and U87-sh-1068-transplanted mice after DMSO and AMD3100 intratumoural injection. Strikingly, our FACS results revealed that there was an almost complete loss of chemotaxis by CD11b^+^Gr-1^+^ MDSCs in AMD3100-treated tumours (DMSO group: U87-sh-NC vs. U87-sh-554, *p* < 0.01; U87-sh-NC vs. U87-sh-1068, *p* < 0.01; AMD3100 group: U87-sh-NC vs. U87-sh-554 vs. U87-sh-1068, n.s. non-significant difference) (Fig. 5D). The increase in the Mo-MDSCs population by downregulation of BATF2 was also rescued by AMD3100 injection (Fig. 5E). Gr-1 and CD11b co-staining in intracranial tumours of the DMSO or AMD3100 groups confirmed the above results (Fig. 5F). Furthermore, our ELISA data indicated that the increased VEGFA, MMP2, and MMP-9 levels upon BATF2 downregulation were significantly impaired after AMD3100 injection (Fig. 5G), suggesting that these tumour-promoting cytokines associated with MDSCs were also blocked by AMD3100. Furthermore, we also performed invasion assays combining EVs and AMD3100, and the results showed that EVs derived from sh-BATF2 cells promoted MDSCs recruitment, compared to the NC group. However, pre-incubation EVs from each group with neutralising anti-SDF-1α antibody (1 μg/mL) or AMD3100 (1 μg/mL) rescued the MDSCs chemotaxis in vitro (Fig. S8D). Taken together, BATF2 inhibits tumour growth and MDSCs recruitment most likely through a decrease of the SDF-1α/CXCR4 signalling. Consequently, blocking the CXCR4 signalling with AMD3100 reverse the effects of the BATF2 downregulation.

### Exo-counter detection identifies BATF2^+^ EVs in plasma as a novel biomarker for glioma

**Fig. 6:**
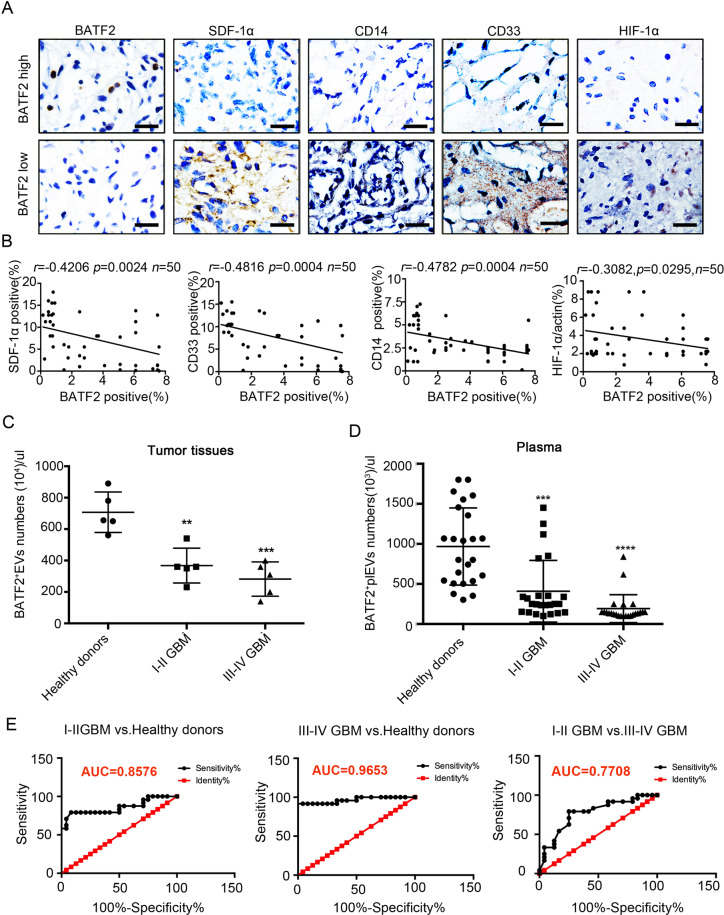
Exo-counter detection identifies BATF2^+^EVs in plasma as a novel biomarker of glioma. **A** Representative images of BATF2, CD33, CD14, SDF-1α, and HIF-1α in human malignant glioma tissues from 50 patients. Scale bars, 100 μm. **B** Correlations analyses of BATF2, CD33, CD14, SDF-1α, and HIF-1α in human malignant glioma tissues from 50 patients. The Pearson’s correlation coefficient (*r*) and *p* value are shown (*n* = 50). **C** Exo-Counter detection of BATF2^+^EVs in tumour tissues of I–II GBM, III–IV GBM and healthy donors (*n* = 5, *p < 0.05, **p < 0.01, ***p < 0.001). **D** Exo-Counter detection of BATF2^+^EVs in plasma of I-II GBM, III-IV GBM and healthy donors (*n* = 24, *p < 0.05, **p < 0.01, ***p < 0.001). **E** ROC curve of BATF2^+^EVs numbers data from I–II GBM vs. healthy donors, III–IV GBM vs. healthy donors, I–II GBM vs. III–IV GBM (*n* = 24, *p < 0.05, **p < 0.01, ***p < 0.001).

To determine whether our findings are clinically relevant, we stained glioma tissue sections (*n* = 50) for BATF2, SDF-1α, HIF-1α (hypoxia marker), CD14 (Mo-MDSCs marker), and CD33 (MDSCs marker). IHC staining showed that BATF2 protein expression was inversely correlated with the expression of SDF-1α (*r* = −0.4206, *p* < 0.001), CD33 (*r* = −0.4816, *p* < 0.001), HIF-1α (*r* = −0.3082, *p* < 0.05), and CD14 (*r* = −0.4782, *p* < 0.001) (Fig. 6A, B). These observations consistently suggested that HIF-1α/SDF-1α plays a role in the inhibition of MDSCs recruitment by BATF2 in glioma tissues. Tumour-derived EVs can enter the systemic circulation with peripheral blood, and it has been shown that EVs in plasma have an effect on tumour progression [25, 34]. Higher levels of EVs are associated with lower MDSCs infiltration, which in turn inhibits glioma progression [1, 4, 9]. Hence, we next sought to calculate BATF2^+^ EV abundance in tumour tissues and plasma in glioma patients and in healthy donors by quantitative Exo-Counter detection. Detailed clinical data are summarised in Table 1. We found that calculating BATF2^+^ EV numbers in tumour tissues can distinguish stages I–II and III–IV GBM patients from healthy donors (I–II GBM vs. healthy donors: *p* < 0.01; III–IV GBM vs. healthy donors: *p* < 0.001) (Fig. 6C). Strikingly, exo-counter-based quantification of the numbers of BATF2^+^ EVs in plasma can also discriminate healthy donors and stage I–II GBM from stage III–IV GBM patients (Fig. 6D). In concordance with the above data, we found a significant inverse correlation between the numbers of BATF2^+^ EVs in plasma and glioma stages, i.e., lower numbers of BATF2-positive EVs in plasma was associated with more advanced stages of glioma. Receiver Operating Characteristic (ROC) analysis showed that BATF2^+^EV detection in plasma showed an Ander Under the Curve (AUC) of 0.8576 (95% CI: 0.7450–0.9703) in stage I–II GBM vs. healthy donors, an AUC of 0.9653 (95% CI: 0.9147–1.016) in stage III–IV GBM vs. healthy donors, and an AUC of 0.7708 (95% CI: 0.6357–0.9059) in stage I–II GBM vs. stage III–IV GBM (Fig. 6E). Collectively, BATF2 protein status can provide a GBM-specific liquid biopsy that can help evaluate glioma staging.

**Table 1:** Clinical characteristics of patients included in plasma EVs detection.

Number of patients	48
Median age at diagnosis	(19–75)
Male:Female ratio	1.2:1
Histologic grade (%)	
WHO grade I-II	24 (50%)
WHO grade III-IV	24 (50%)
Molecular diagnosis (%)	
Glioblastoma	18 (37.5%)
Oligodendroglioma	18 (37.5%)
Astrocytoma	12 (25%)
Extent of resection (%)	
Gross total resection	6 (12.5%)
Subtotal resection	6 (12.5%)
Biopsy	36 (75%)
Adjuvant treatment (%)	
RT only	9 (18.75%)
RT with chemotherapy	0
Chemotherapy only	0
None	39 (81.25%)
Median follow-up	
Non-progressors	6 years
Survivors	5 years
IDH1/2	
+	25 (52%)
−	23 (48%)

## Discussion

Crosstalk between tumour cells and the tumour microenvironment has attracted great attention from researchers in the field [41, 42]. BATF2 is a nuclear transcription factor that contacts AP-1 to exert tumour-suppressive effects [28]. In our study, we found that BATF2 effectively inhibited tumour growth. However, overexpression of BATF2 did not affect tumour cell proliferation and migration, but leading to decreased MDSCs infiltration in tumour tissues (Fig. 2A, B). Indeed, our data confirmed that higher expression of BATF2 can be detected in EVs released from BATF2-overexpressing glioma cells (Fig. 3C). Moreover, EVs from BATF2-overexpressing glioma cells inhibited MDSCs recruitment in vitro (Fig. 3E). These data may provide evidence that BATF2-bearing EVs may participate in GBM inhibition caused by BATF2. At the same time, additional studies indicate that the release of EV contents driven by specific oncogenes are functionally transferred to target cells via EVs [26, 43]. However, whether manipulation of BATF2 expression leads to overall changes in EV contents, and hence their effects on MDSCs infiltration, remains unclear and warrants further studies. Investigations into EVs contents and pathways could provide a more detailed understanding of the effects of BATF2 on the contents of EVs.

However, data from https://www.proteinatlas.org show that BATF2 is located in both the nuclear membrane and nucleolus. In our study, we were the first to identify that BATF2 could be detected in EVs, and that BATF2 protein on the EVs surface could be labelled with gold-conjugated anti-BATF2 antibody and scanned by TEM (Fig. 3G), which may contribute provide further evidence to BATF2^+^ EV detection by exo-counter. However, the topological structure of BATF2 loaded onto the EVs is not clear and warrants further study.

A previous study reported that high CD33 levels confer a poor prognosis for GBM patients [43]. In our study, high CD33 and low BATF2 expression were observed in tissue samples from GBM patients in advanced clinical stages. MDSCs are divided into two distinct subsets based on the expression of two molecules, Ly-6C and Ly-6G [6]. In our study, we found that BATF2 mainly inhibits monocyte-derived MDSCs (Mo-MDSCs), defined as CD11b^+^Ly-6G^low^Ly-6C^high^. We also found that high CD14 (Mo-MDSC marker) levels confer poor prognosis in GBM patients with low BATF2 expression levels. In addition to the decrease in MDSCs infiltration, low MMP-9, VEGFA, and MMP2 expression was observed in the BATF2 group. These cytokines are the main MDSCs-secreted factors that promote angiogenesis, tumour growth, and invasion [43, 44].

It is possible that soluble factors, including CCL2, SDF-1α, M-CSF, GM-CSF, and G-CSF, are secreted by tumour cells [8, 10, 45]. To assess the effects of BATF2 on inhibition of MDSCs infiltration, ELISA was performed to evaluate the expression levels of five chemokines. These factors mainly regulate MDSCs recruitment and expansion in the tumour microenvironment [34]. SDF-1α is widely expressed in various organs such as the lungs, liver, skeletal muscles, brain, kidneys, heart, skin, and bone marrow [46]. Its primary function is involved in the homing of hematopoietic stem cells to bone marrow [46, 47]. Multiple lines of evidence indicate that SDF-1α promotes proliferation and survival of tumour cells in ovarian cancer [48], prostate cancer [49], acute myeloid leukaemia [46], and GBM [15]. In addition, SDF-1α has been previously reported as a chemo-attractant for MDSCs that express cognate receptors such as CXCR4 and CXCR7 [50]. We observed that BATF2 inhibit both HIF-1α and SDF-1α expression in U251 cells and tumour tissues (Fig. 4G). HIF-1α pathways have been reported to increase SDF-1α expression [12, 37, 38, 46]. We hypothesise that BATF2 represses HIF-1α transcriptionally (this has been shown by Dai et al. in CRC model [33]), and this in turn prevents transcriptional activation of SDF-1α by HIF-1α, leading to downregulation of SDF-1α. SDF-1α reduction was observed in intracellular in U251-BATF2 cell (Supplementary Fig. 8A, B). Besides, reduced SDF-1α observed in U251 after BATF2-EV uptake depended on HIF-1α (Fig. 4H–J). However, the main mechanisms responsible for BATF2-EVs induced inhibition of SDF-1α was not very clear and needed to study.

There are certain limitations to our study. The TME in GBM is complex due to native extrinsic components as well as tumour intrinsic mechanisms [7, 14]. We focused on bone marrow-derived cells (CD45^+^cells, mainly including MDSCs and TAMs). However, the effects of BATF2 on other cellular components of the brain microenvironment, such as astrocytes, neurons, and endothelial cells, are worth studying.

In conclusion, we found that BATF2 inhibits MDSCs infiltration and glioma growth. EVs from BATF2 upregulated cells could inhibit MDSCs recruitment in vitro. Moreover, BATF2 inhibited intracellular SDF-1α, and also contributed to decreased SDF-1α loading on EVs. Thus, by combining AMD3100 to block SDF-1α/CXCR4 signalling, the promoting effects of BATF2 downregulation on MDSCs recruitment were rescued. Furthermore, by calculating the numbers of BATF2-positive EVs in plasma, we proposed the clinical use of BATF2^+^plEVs as a biomarker to reflect glioma stage.

## Materials and methods

### Cell lines and cell culture

All glioma cell lines (LN-18, U87-MG, U251, A172, and U118-MG) and human astrocytes were purchased from American Type Culture Collection (ATCC, Manassas, VA, USA) and cultured in Dulbecco’s modified Eagle’s medium (DMEM) supplemented with 10% FBS.

### EV concentration

EVs from cell culture supernatants were isolated by ultracentrifugation. Briefly, cells were grown in DMEM medium with exosome-depleted 5% FBS (Gibco Thermo Fisher Scientific). The media (~100 mL) was collected and pre-cleared by centrifugation at 500 × *g* for 10 min at 4 °C, then the supernatant was removed and discarded. After suspension in PBS, a second round of centrifugation at 2500 × *g* for 20 min at 4 °C to remove cell debris was performed. The supernatant was filtered through a 0.22 μm filter to remove larger microvesicles and then ultracentrifuged at 135,000 × *g* for 120 min (Optima XPN-100 ultracentrifuge, Beckman Coulter) using a Beckman Ti70 fixed-angle rotor. The EV pellet was washed with PBS followed by a second step of ultracentrifugation at 135,000 × *g* for 2 h at 4 °C, as recommended by MISEV2018 [42].

### EV characterisation

EVs were evaluated for morphology by TEM. EVs were resuspended in PBS and 50 μL of exosomes was absorbed onto Formvar (Polysciences, Inc.) carbon-coated nickel grids for 1 h. Then, the grids were sequentially washed with 0.1 M sodium cacodylate, pH 7.6, fixed in 2% para-formaldehyde and 2.5% glutaraldehyde in 0.1 M sodium cacodylate and contrasted with 2% uranyl acetate in 0.1 M sodium cacodylate for 15 min. After another wash, grids were incubated with 0.13% methyl cellulose and negatively stained with 0.4% uranyl acetate for 10 min, air-dried, and visualised under a JEM-2200FS transmission electron microscope operated at 100 kV.

### Nanoparticle tracking analysis (NTA)

A NanoSight NS300 system (Malvern, Worcestershire, UK) was applied to determine the size and concentration of particles and confirm that their sizes were equivalent to those of EVs. A total of five videos, 30 s each, were recorded for individual samples. EVs were resuspended in PBS at a concentration of 2 μg/μL (1:100 dilution in particle-free PBS) to achieve 5 × 10^7^−5 × 10^9^ particles/mL. Samples were manually injected into the sample chamber at ambient temperature. Each sample was configured with a blue 405 nm laser and a high-sensitivity scientific complementary metal-oxide semiconductor (sCMOS) camera. At least 200 completed tracks were analysed per video. Particles were tracked, quantitated, and their sizes determined using NTA software v.3.0.

### Animal study

Thirty micrograms (about 0.56 × 10^6^ particles) of Ctrl-EVs and BATF2-EVs derived from each cell line were mixed in 150 µL of PBS. Fifty microlitres of the EV mix (final amount, 10 μg EVs) was injected intratumourally into U251-bearing mice.

### Intracranial tumour formation in vivo

U87-MG, U251, and GL261 cells were used to establish a tumour model in BALB/c nude mice (6 weeks old, female). After 5 weeks, the animals were euthanised, dissected, and subjected to MRI, micro-CT, and H&E staining. All animal experiments were approved in accordance with the institutional guidelines of the Ethics Committee of West China Hospital, Sichuan University.

### Antibodies

Antibodies used in this study were as follows: BATF2 (ab157466, Abcam, London, UK); BATF2 (Thermo Fisher, PA5-43094); BATF2 (241887, Santa Cruz Biotechnology, CA, USA); CD63 (Abcam, ab217345); CD9 (Abcam, ab92726); TSG101 (Abcam, ab125011); SDF-1α (Abcam, ab9797); SDF-1α (Abcam, ab181018); HIF-1α (H6536, Sigma); CXCR4 (Abcam, ab135170); VEGF (ab46154, Abcam); CD33 (Abcam, ab203253); CD14 (Abcam, ab203294); Gr-1 (108448, Biolegend); CD11b (Abcam, ab13357); CXCR7 (Biolegend, #391405); SV40 Large T Antigen (CST, #15729, CA, USA); MMP-9 (CST, #13667); 10 nm gold-conjugated goat anti-rabbit secondary antibody (bs-0437P-Gold, Bioss, China), GAPDH (sc365062, Santa Cruz Biotechnology), and β-Actin (sc-47778, Santa Cruz Biotechnology).

### Transwell invasion assay of MDSCs

To examine the chemotactic ability of glioma cells, freshly isolated splenic MDSCs were seeded on the upper chambers of 24-well transwell inserts (Merck Millipore). In the bottom chambers, tumour-derived EVs from cell supernatant were added. A total of 7 × 10^3^ Ctrl-EV and BATF2-EV/target MDSC which was found effective in preliminary experiments performed with different EV numbers ranging from 5 × 10^3^ to 1 × 10^4^. Invasive MDSCs accumulated at the lower chambers were collected after 16 h and counted using a cytometer.

### Statistical analysis

All experiments were repeated 3–5 times, and error bars are presented as means ± s.d. Statistical significances were calculated by using Student’s *t* test in either pairwise or multiple comparisons; *p* < 0.05 was considered statistically significant. For quantification of IHC staining, Mann–Whitney *U* tests were used to calculate *p* values. Spearman rank-order correlation coefficient analysis was performed to assess relationships between different factors. All calculations were performed using SPSS v.17.0 software (SPSS Inc., Chicago, IL, USA) or Prism GraphPad 7.0.

## Supplementary information

movieS1

Supplementary Figures
