# Supplementary material for: BATF2 prevents glioblastoma multiforme progression by inhibiting recruitment of myeloid-derived suppressor cells
Source: Oncogene. 2021 Jan 15;40(8):1516–30. doi: 10.1038/s41388-020-01627-y (PMC7906906; doi:10.1038/s41388-020-01627-y)
Supplement: Supplementary file 2 — Supplementary Figures [file 41388_2020_1627_MOESM2_ESM.docx]

**Supplementary Figure 1**

**
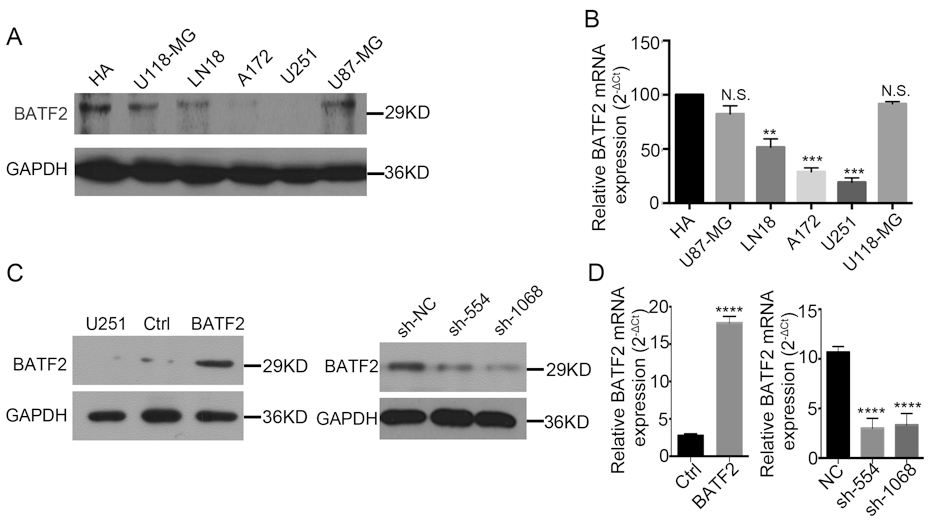
**

A-B. Basal BATF2 protein and mRNA expression levels in glioma cell lines and astrocyte cell line detected by western blotting.

C-D. Western blot and q-PCR showing BATF2 expression in glioma cells and astrocyte cell line. (n=3 independent experiment, * *p*<0.05, ** *p*<0.01, *** *p*<0.001)

**Supplementary Figure 2**


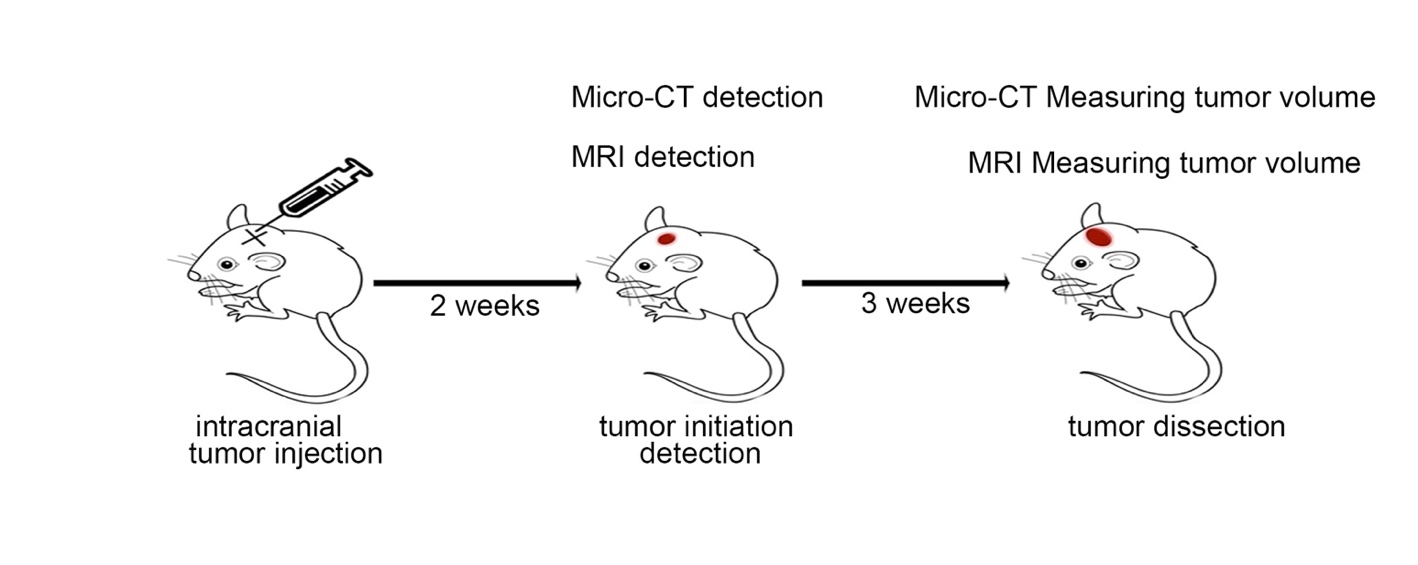


1. Experimental design for MRI or Micro-CT detection in glioma-bearing mice.

**Supplementary figure 3**

**
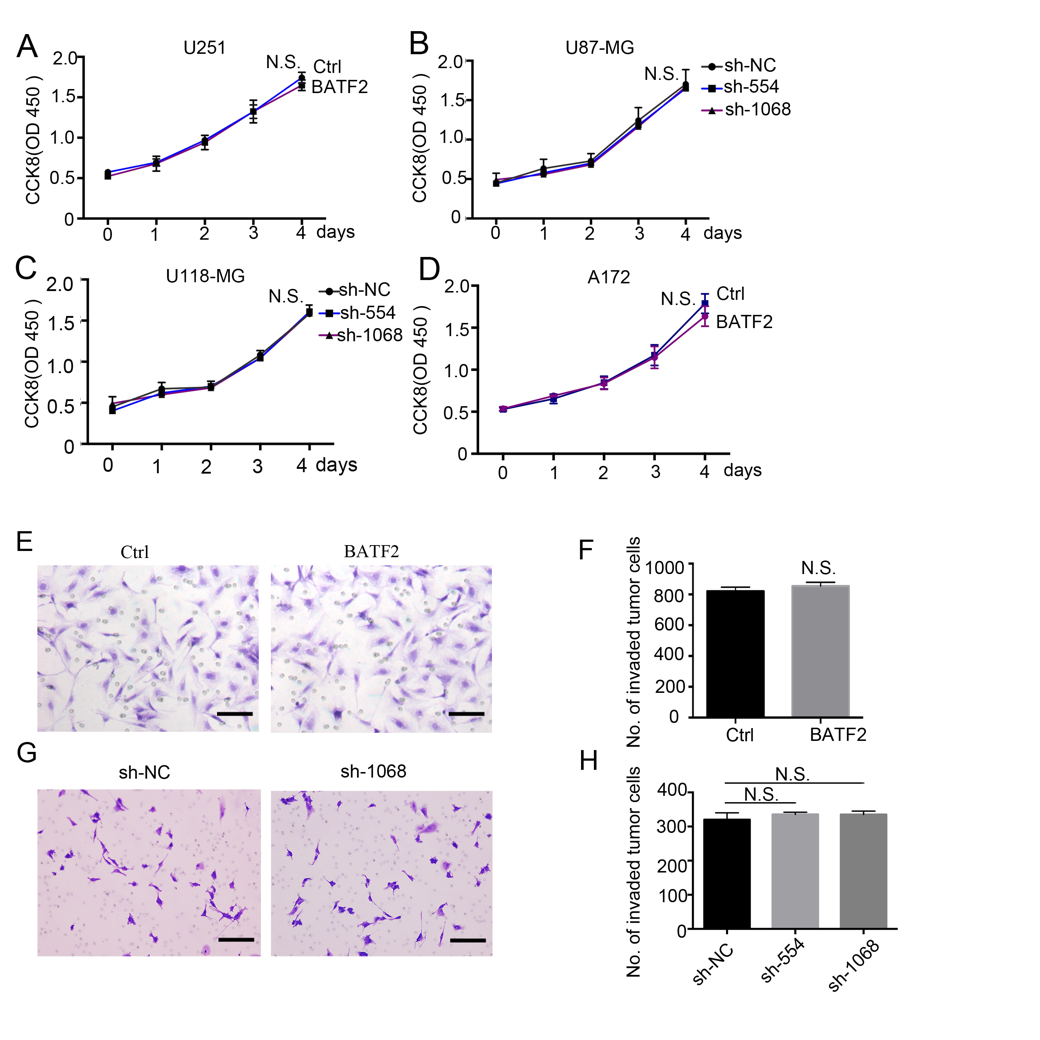
**

A-D. Cell proliferation of U251, U87, U118, A172 group was determined by CCK8 assay. (n=3, independent experiment, N.S., non-significance)

E-F. Invasive properties and statistics of U251-Ctrl and U251-BATF2 were determined with invasion assays using Matrigel invasion chambers. Representative results are shown. (n=3, independent experiment, N.S., non-significance)

G-H. Invasive properties and statistics of U87-sh-NC, U87-sh-554, and U87-sh-1068 were determined with invasion assays using Matrigel invasion chambers. Representative results are shown. (n=3, independent experiment, N.S., non-significance)

**Supplementary Figure 4**

**
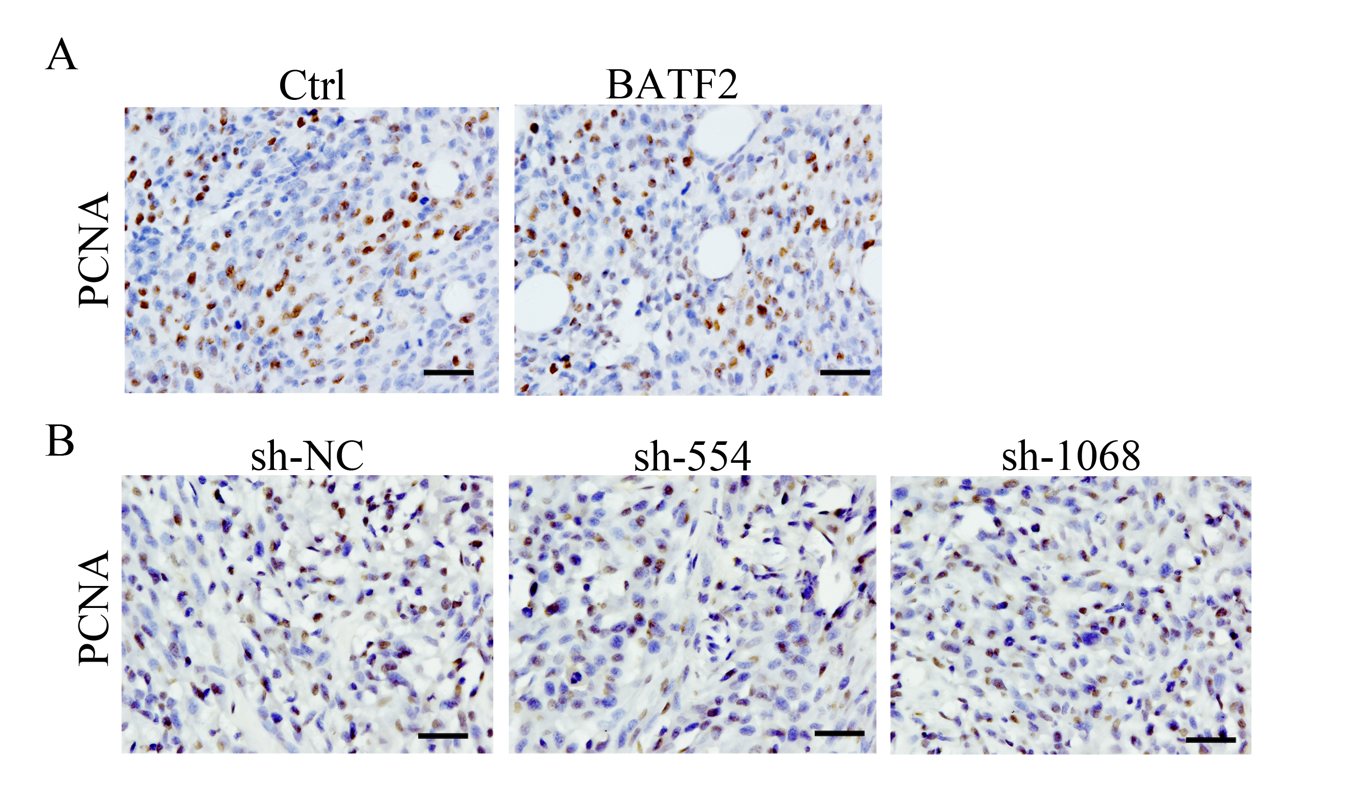
**

1. Representative IHC images of PCNA staining on consecutive sections of U251-Ctrl, U251-BATF2. Scale bars,20μm.
2. Representative IHC images of PCNA staining on consecutive sections of U87-sh-NC, U87-sh-554, and U87-sh-1068. Scale bars,20μm.

**Supplementary Figure 5**


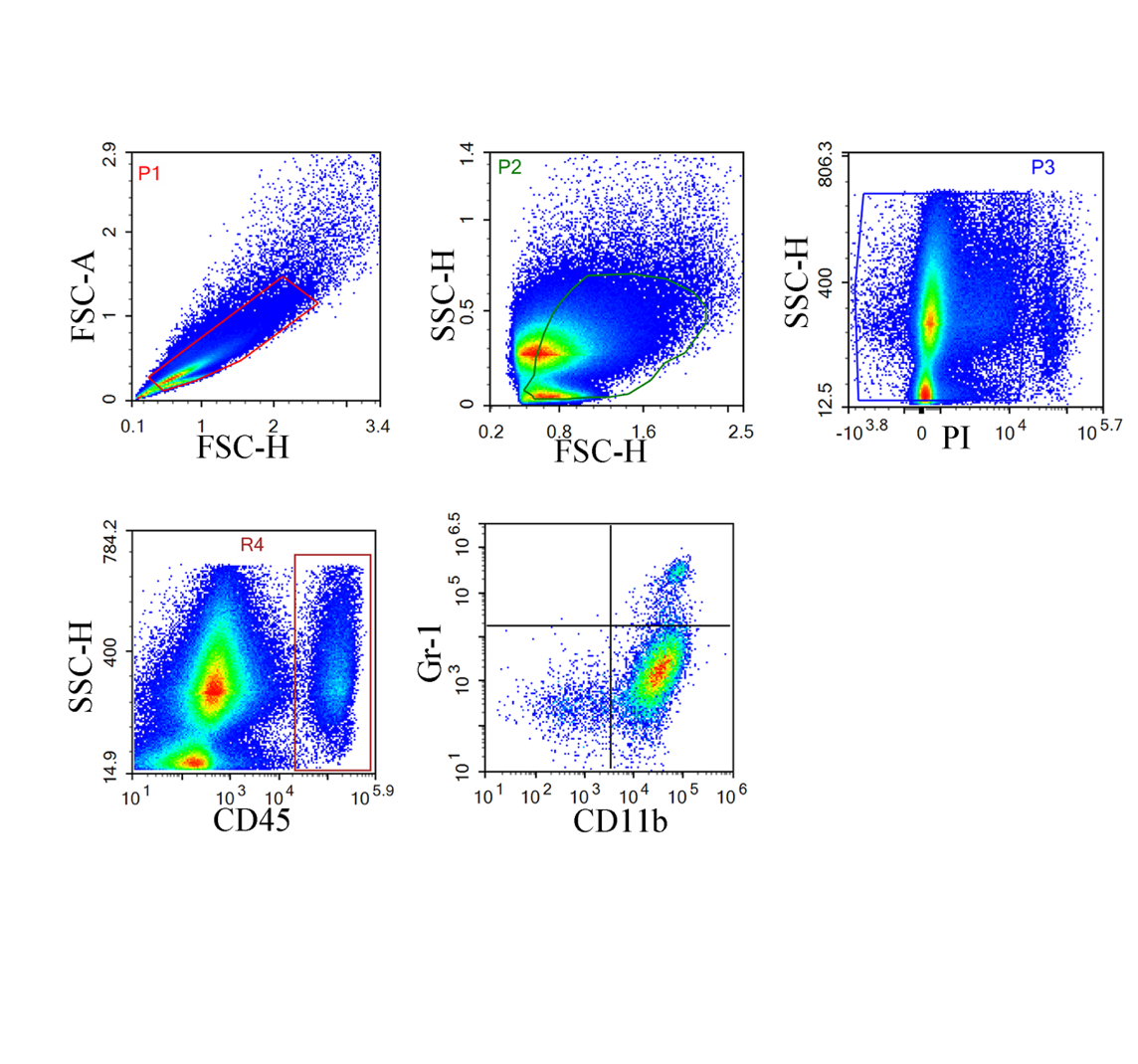


1. Experimental design for MDSCs analysis by FACs in glioma bearing mice.

**Supplementary Figure 6**

**
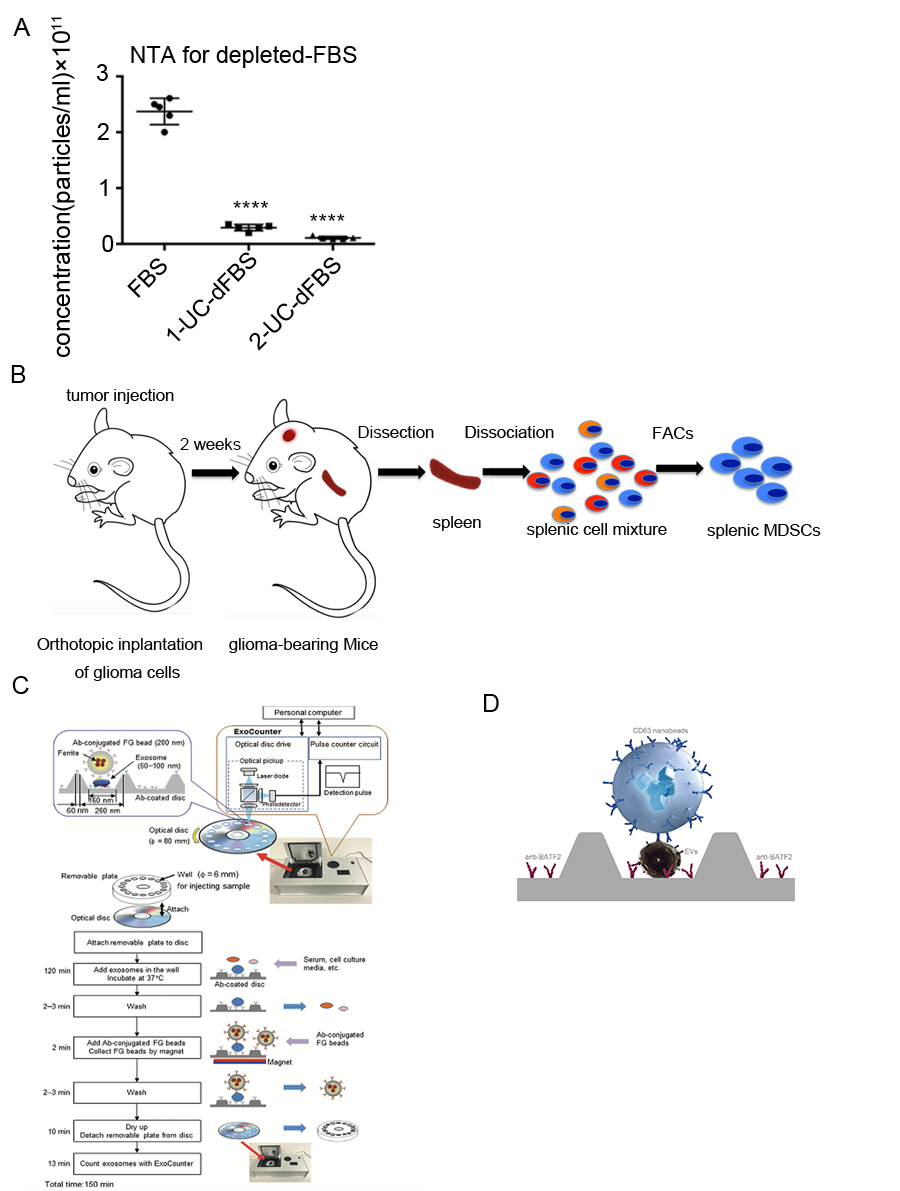
**

1. EVs concentration and size distribution by nanoparticle tracking analysis (NTA). Concentrations (particles/ml of original FBS) are shown in the y-axis and the EVs-depleted FBS and regular FBS samples on the x-axis. The UC-dFBS (ultracentrifugation-depleted EVs FBS）contained fewer particles than the regular FBS. The dots depict measurements from technical replicates. (n=5, independent experiment, **** *p*<0.0001)
2. Experimental design for MDSCs sorting in glioma-bearing mice.
3. Schematic overview of the Exo-Counter ^(22)^
4. BATF2 antibody coating to the disc combining to CD63 nanobeads detection platform.

**Supplementary Figure 7**


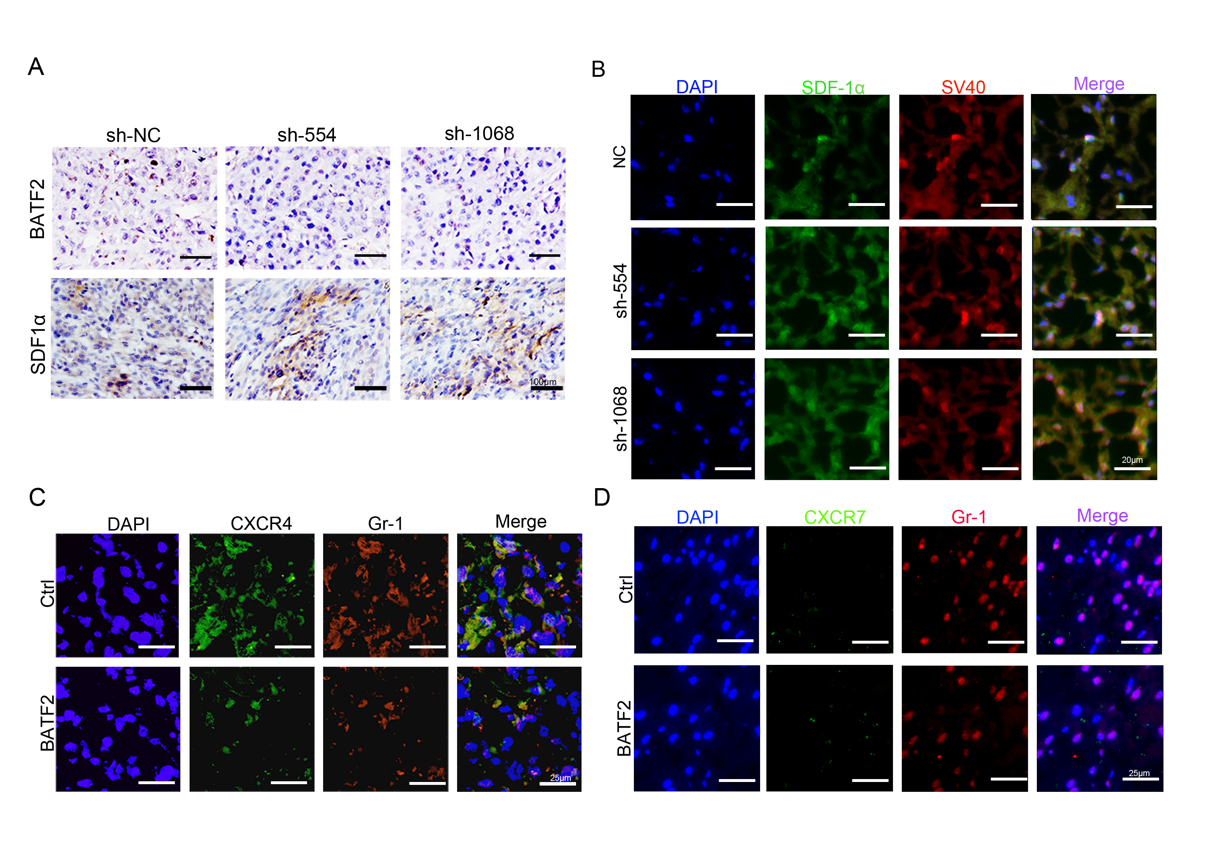


1. Representative IHC images of BATF2 and SDF-1α on consecutive sections of U87-sh-NC, U87-sh-554 and U87-sh-1068 intracranial tumors. Scale bars,100μm.
2. Representative image of immunofluorescent staining with SDF-1α (green), SV40 large T (red) and DAPI (blue) in U87-sh-NC, U87-sh-554, U87-sh-1068 intracranial tumors. Scale bars, 20μm.
3. Representative image of immunofluorescent staining with CXCR4 (green), Gr-1 (red) and DAPI (blue) in U251-Ctrl and U251-BATF2 intracranial tumor frozen sections. Scale bars, 25μm.
4. Immunofluorescent staining of CXCR7 (green), Gr-1 (red) and DAPI (blue) in U251-Ctrl and U251-BATF2 intracranial tumor frozen sections. Scale bars, 25μm.

**Supplementary Figure 8**

1. Western blotting detection of HIF-1α and GAPDH in U251-Ctrl and U251-BATF2.
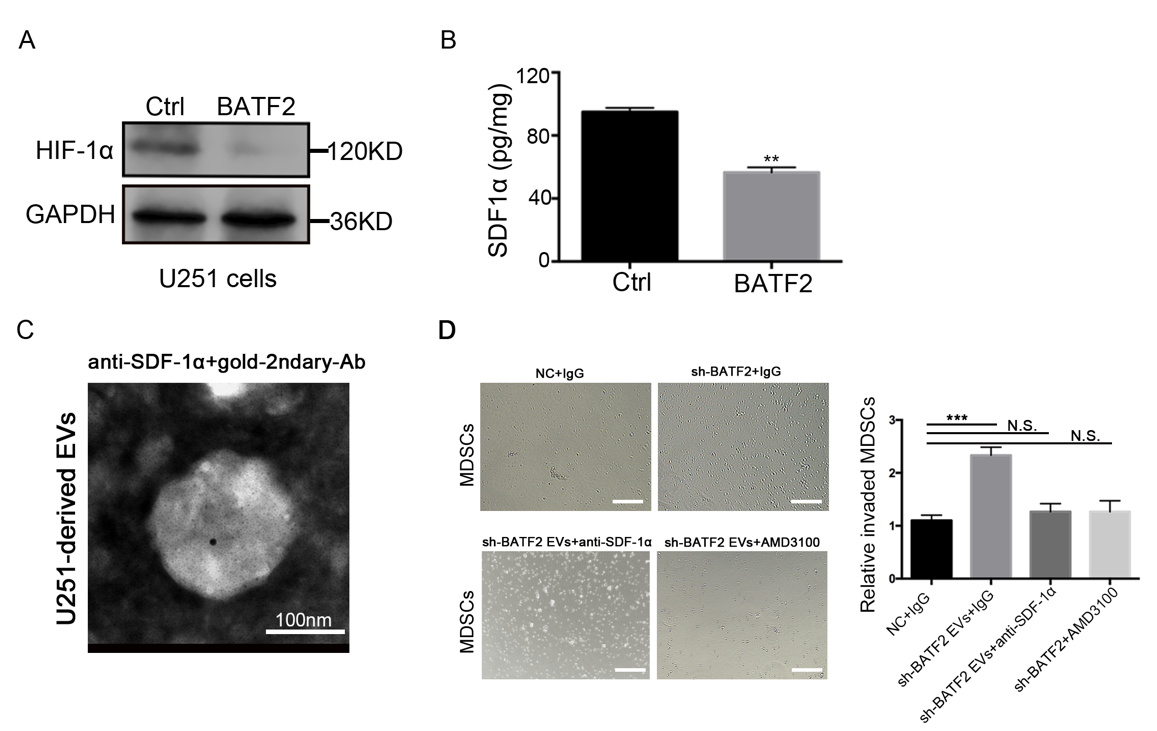

2. Elisa detection of SDF-1α in U251-Ctrl and U251-BATF2. (n=3, independent experiment, * *p*<0.05, ** *p*<0.01, *** *p*<0.001)
3. Transmission electron microscopy of U251-derived EVs stained with primary anti-SDF-1α and gold-conjugated secondary antibody. Arrow indicates EV staining positive for SDF-1α. Scale bar, 100 nm.
4. Representative images and statistics of invaded MDSCs cells after co-culture with U87-NC、U87-shBATF2、U87-shBATF2+antiSDF-1α、U87-shBATF2+AMD3100 derived EVs. Scale bars, 200μm (n=3, independent experiment, NS, non-significance, *** *p*<0.001)

**Movie S1**

3D micro-CT concluded glioma model established. (mp4. File)

**Supplementary Materials and Methods**

**Animals**

Balb/C nude female mice of 6-week-old were purchased from the Model Animal Research Center of Nanjing University (Nanjing, China) and allowed to acclimate for 1 week before use. All mouse care and experiments were carried out in accordance with institutional guidelines concerning animal use and care of Sichuan University. Six mice per group were injected subcutaneously (s.c.) with U87-sh-NC、U87-sh-554、U87-sh-1068 cells per flank of mice. Tumor size was determined by collecting length and width measurements and calculating the tumor volume (mm3) as (tumor length × (tumor width)2) × 0.52. When mice were killed, tumors from each animal were collected, weighed and data were expressed as mean ± SD, n= 6.

**Lentivirus-BATF2**

Human BATF2 plasmids were obtained from Origene (Rockville, MD, USA) and cloned into the pHBLV-CMVIE-IRES-puro lentiviral vector (Neuron Biotech, Shanghai, China). Mouse BATF2 plasmids were obtained from Origene (Rockville) and cloned into the pHBLV-CMV-MCS-3flag-EF1-puro lentiviral vector (Hanbio Co. LTD, Shanghai, China).

**Flow cytometry**

MDSCs were stained with the following antibodies: CD45-APC, Gr-1-FITC, F4/80-FITC, CD11b-percp/cy5.5, and CXCR7-APC-Cy7 (eBioscience); CXCR4-PE (BD Biosciences). CD45^+^ populations were analyzed for the expression of Gr-1, F4/80, and CD11b. Stained cells were measured on FACS Aria flow cytometer (Becton Dickinson, NJ) and analyzed by FlowJo software. The experiments were repeated thrice using 6 independent tumor samples for each combination of markers.

**Isolation of spleen cells**

Splenocytes were isolated from the spleens of tumor-bearing mice by disaggregation into 10 mL of RPMI 1640 complete medium. Erythrocytes were lysed with Red Blood Cell Lysis Buffer (Beyotime, Nanjing, China). Then, splenocytes were mashed through 70-μm cell strainers to obtain single-cell suspensions.

**Isolation of MDSCs by FACs sorting**

For flow cytometric sorting, 1×10^7^ cells/mL splenocytes from tumor-bearing mice were stained with anti-CD11b-APC (BD Biosciences), anti-Ly6G-FITC (BD Biosciences), and anti-Ly6C-PE (BD Biosciences) antibodies for 20 min on ice in staining buffer (1% FBS in PBS). Cells were then washed with PBS, and the samples were then sorted using a BD Influx. To obtain pure cells, 1.5 drop pure sort mode was chosen. The cells were sorted by gating on P1 (CD45^+^ populations) and P2 (CD45^+^CD11b^+^Gr-1^+^ populations).

**ELISA**

The protein levels of SDF-1α, MMP-9, VEGF, GM-CSF, CCL2, M-CSF, and G-CSF in all groups of U251 and U87-MG intracranial tumors from mice were detected using their respective ELISA kits (NeoBioscience).

**Multi-center clinical sample collection**

Plasma of GBM patients and healthy donors were collected from Nanfang Hospital. 50 pairs of GBM tissues were collected from Huaxi Biobank (West China Hospital, Sichuan University, Chengdu, Sichuan). The collection and application of clinical samples were approved by the Ethics Committee of West China Hospital of Sichuan University and Nanfang Hospital of Southern Medical University.

**EVs detection by Exo-counter**

Exo-counter could detect specific EVs derived from cells, tissues or human plasma without any enrichment procedures. The optical discs were made using standard manufacturing equipment for DVDs or Blu-ray discs. The optical disc was attached with a removable plate containing 16 wells for sample injection. Each well was coated with 5mg/L anti-BATF2 antibody in carbonate-bicarbonate buffer (PH9.6) overnight at 4°C. Next, 12.5μl plasma in 37.5 μl PBS of sample solution was added to each well and incubated for 2h at 37°C followed by washing with PBST^22^.

**Nano-flow cytometry**

Two single-photon counting avalanche photodiodes were used for the simultaneous detection of the side scatter (SSC) and fluorescence of individual EVs, respectively. Each distribution histogram or dot-plot was derived from data collected 1 min. 30 μg EVs suspension was diluted to 100 μL with PBS and blocked with 2% BSA with rotation at room temperature for 30 min and incubated with anti-SDF-1α-FITC. Labeled EVs were washed with PBS twice by ultracentrifugation and resuspended in 50 μL PBS for nFCM analysis. The percent positive particle was calculated relative to the total number of particles analyzed per sample^46^. This percentage was therein referred to as the percent with SDF-1α^+^EVs.

**GBM patients’ plasma collection**

Blood samples included in this study (detailed clinical data are summarized in **Table 1)** were collected in 10 mL vacutainer tubes with EDTA anticoagulant (REF367525; BD, USA). The tubes with blood samples were gently inverted eight times to mix, stored upright, and then shipped at 4°C within 1 h after collection. To harvest plasma, blood samples were centrifuged at 1500×g for 15 min at 4°C and each 1 mL fraction of the supernatant was transferred into a fresh 1.5 mL tube and stored at -80°C.

**PKH67 labeling of EVs**

The PKH67 Green Fluorescent Cell Linker Mini Kit for General Cell Membrane Labeling kit (SLBR4806V, Sigma) was used to label EVs. Briefly, isolated EVs (~107) from cells were resuspended in 100μl of diluent C and then mixed with 2μM of PKH67 dye (diluted in diluent C), followed by 5 mins incubation at room temperature. Then the reaction was stopped by adding 500 μl of 1% BSA (bovine serum albumin)/PBS. Finally, labeled EVs were washed twice with PBS followed by ultracentrifugation and resuspension in PBS.

**EVs gold labelling**

EVs were fixed by 2% PFA for 20-30 min before absorbed on the carbon-coated copper grids for 20 min and then washed by PBS for 3 times. Next, EVs were blocked with 5% BSA for 10min, washed with PBS and incubated with BATF2 antibody for 30 min, followed by PBS washing. Next, EVs were incubated with 10 nm of secondary antibody for 30min, then washed by distilled water and finally embedded in 2% phosphate-tungstic acid.

**Micro-CT procedure**

Animals were scanned using a Micro-CT scanner (J Morita Engineering Co., Ltd, Japan). This *in vivo* Micro X-ray CT system consists of an X-ray tube (X-ray energy, max 90 kV), semiconductor detector with 200 × 200 μm pixel size, and the X-ray voltage and current were set at 90 kVp and 50 μA, respectively.

**MRI detection of orthotopic glioma**

Intracranial glioma growth was monitored *in vivo* in tumour-bearing mice by MRI after inoculation using a Bruker 7.0T scanner (Bruker BioSpin GmbH, Germany). T_2_-weighted images were acquired using a rapid acquisition relaxation-enhanced sequence^52^.

**Immunostaining and histology**

Glioma patients (age: 55–75 years; clinical glioma tissue grade: WHO classification I–IV) were recruited, and biopsies were performed at West China Hospital (Chengdu, China). This study was approved by the Ethics Committee of West China Hospital. Positive staining extent of proteins was calculated using ImageJ software. Five microscopic fields were randomly chosen for each image. The BATF2 positive rate was higher than the median positive percentage considered to be BATF2 high and lower than the median considered low expression. Correlations involving proteins in human glioma tissue samples were analysed using Prism Graphpad 7.0.

**Western blotting**

EVs were homogenised in 100 μL RIPA lysis buffer with protease inhibitors (P0013B; Beyotime, Shanghai, China) on ice for 30 min. Samples were then centrifuged at 12,000 × *g* for 10 min at 4°C, and 80 μL supernatant was combined with 20 μL 5 × SDS-PAGE Sample Loading Buffer (P0015; Beyotime). The protein concentration of concentrated EVs was measured using BCA assays according to the manufacturer’s instructions. The mixtures were then boiled for 10 min at 100°C. 40 μg of protein samples was separated via SDS-PAGE on 4-20% gels (Bio-Rad, Redmond, WA, USA), electroblotted onto polyvinylidene difluoride membranes (Millipore, Billerica, MA, USA), and then incubated with primary antibody at ambient temperature for 2 h. Antibody binding was detected using an enhanced chemiluminescence system in accordance with the manufacturer’s protocol (Tanon-5200 Multi; Shanghai, China). Protein levels were calculated from three independent experiments using western blotting.
